# Understanding the pathogenesis of multiple system atrophy: state of the art and future perspectives

**DOI:** 10.1186/s40478-019-0730-6

**Published:** 2019-07-12

**Authors:** Giacomo Monzio Compagnoni, Alessio Di Fonzo

**Affiliations:** IRCCS Foundation Ca’ Granda Ospedale Maggiore Policlinico, Dino Ferrari Center, Neuroscience Section, Department of Pathophysiology and Transplantation, University of Milan, Via Francesco Sforza 35, 20122 Milan, Italy

**Keywords:** Multiple system atrophy, Pathogenesis, Alpha-synuclein, Mitochondria

## Abstract

Multiple System Atrophy (MSA) is a severe neurodegenerative disease clinically characterized by parkinsonism, cerebellar ataxia, dysautonomia and other motor and non-motor symptoms.

Although several efforts have been dedicated to understanding the causative mechanisms of the disease, MSA pathogenesis remains widely unknown.

The aim of the present review is to describe the state of the art about MSA pathogenesis, with a particular focus on alpha-synuclein accumulation and mitochondrial dysfunction, and to highlight future possible perspectives in this field.

In particular, this review describes the most widely investigated hypotheses explaining alpha-synuclein accumulation in oligodendrocytes, including *SNCA* expression, neuron-oligodendrocyte protein transfer, impaired protein degradation and alpha-synuclein spread mechanisms.

Afterwards, several recent achievements in MSA research involving mitochondrial biology are described, including the role of *COQ2* mutations, Coenzyme Q10 reduction, respiratory chain dysfunction and altered mitochondrial mass.

Some hints are provided about alternative pathogenic mechanisms, including inflammation and impaired autophagy.

Finally, all these findings are discussed from a comprehensive point of view, putative explanations are provided and new research perspectives are suggested.

Overall, the present review provides a comprehensive and up-to-date overview of the mechanisms underlying MSA pathogenesis.

## Introduction

Multiple System Atrophy (MSA) is a progressive and severe neurodegenerative disorder which is clinically characterized by variable degrees of parkinsonism, cerebellar ataxia and dysautonomia. Additional motor and non-motor symptoms can be detected as well. Two clinical subtypes, MSA-P and MSA-C, can be distinguished on the basis of the predominant symptomatology, parkinsonian or cerebellar, respectively. [27, 58, 121].

Estimated incidence is 0.6–0.7 per 100,000 person-years and a geographical pattern can be observed in the worldwide distribution of the two subtypes. MSA-P is the most common subtype in Western countries, while a predominance of MSA-C cases is observed in Japan [32, 55, 128].

Onset is usually in the sixth decade of life and prognosis is poor with a mean survival of 6–10 years from the disease onset [27].

Although several pharmacological compounds have been tested and various pre-clinical and clinical therapeutic trials are ongoing, an effective cure is not available yet [123].

Neuropathologically, MSA is characterized by putaminal, pontine and cerebellar atrophy [41]. The complexity of the neuropathological pattern correlates with the spectrum of the clinical phenotypes. Although several overlaps can be observed between MSA-P and MSA-C, each subtype is characterized by specific neuropathological features. MSA-P is denoted by severe striatonigral degeneration. The dorsolateral caudal putamen and the caudate nucleus are severely affected, with a selective involvement of GABAergic medium spiny neurons [97]. Substantia nigra dopaminergic neurons are also remarkably involved in the degenerative process and a trans-synaptic degeneration of striatonigral fibers has been proposed. Globus pallidus and subthalamic nucleus are also implicated [41]. Although signs of striatonigral degeneration can also be observed in MSA-C, this subtype is more severely characterized by the involvement of cerebellar vermis and hemispheres, dentate nucleus, inferior olive nuclei, pontine basis and cerebellopontine fibers [41]. Both MSA-P and MSA-C are characterized by the involvement of other regions of the nervous system, including intermediolateral column of the spinal cord, dorsal nucleus of vagus and Onuf’s nucleus [129]. Motor and supplementary motor cortices are also implicated [119].

Glial cytoplasmic inclusions (GCIs), intracellular protein aggregates mainly composed of α-synuclein (α-syn) and located in oligodendrocytes, are the most important microscopic hallmark of the disease [41, 43, 84, 85]. However, α-syn aggregates can also be detected in neurons, both in cytoplasm (neuronal cytoplasmic inclusions, NCIs) and in nuclei (neuronal nuclear inclusions) [121]. Neuronal loss, axonal degeneration, microglial activation and astrogliosis are other prominent microscopic features of the disease [41].

The relationship between the peculiar oligodendroglial pathology and the neurodegenerative process has been widely investigated. The finding of a positive correlation between neuronal loss and GCI density suggests a possible association [39, 41, 82]. However, some exceptions, including the finding of a severe neuronal loss in the substantia nigra which is not accompanied by a proportionally high GCI burden, still raises some concerns [41, 82].

Although the cause of MSA is still obscure, a relevant effort has been dedicated to understanding the pathogenic mechanisms [44]. This review focuses on α-syn accumulation, which is by far the most widely investigated mechanism, and mitochondrial dysfunction, whose analysis has provided relevant advances in recent years. However, other putative mechanisms, including inflammation [114, 115, 126], autophagic impairment [75, 76, 103, 117], proteasomal dysfunction [16, 112] and iron metabolism dysregulation [49] are also discussed.

## Alpha-synuclein

Most of the studies assessing the pathogenesis of MSA have focused on the mechanisms underlying α-syn intracellular accumulation. Alpha-syn, which plays a crucial role also in Parkinson’s disease (PD) and dementia with Lewy bodies (DLB) [108, 109], is a key protein in MSA neuropathology. The finding of α-syn accumulation not only in neurons, but also in oligodendrocytes, is an important feature of this disease. Furthermore, GCIs, whose presence is required for a diagnosis of “definite MSA” [33], are the main pathological hallmark.

Alpha-syn is a 14 KDa protein, composed of 140 amino acids, which is physiologically expressed in the human brain. Its physiological conformation is not completely clear, being still a matter of debate whether its native conformation is a folded tetramer of 58–60 KDa or an unfolded/disordered monomer which assumes an extended conformation in native gels [7, 28, 61, 127]. The precise function of α-syn is still obscure, although several studies have pointed out a putative role in regulating synaptic vesicles and neurotransmitter release [10]. Furthermore, more complex forms of the protein, in particular oligomers and fibrils [61], and post-translational modifications (e.g. phosphorylation, nitration and ubiquitination) [6, 80] have been associated with synucleinopathies.

As opposed to neurons, healthy mature oligodendrocytes have not been described to express α-syn [107] and the presence of α-syn in oligodendrocyte precursors is still debated because some laboratories have detected a basal level of α-syn expression in non-primate mammals and humans, while others have not [1, 22, 71, 93]. Therefore, the finding of α-syn aggregates in oligodendroglia is even more remarkable.

Several hypotheses have been proposed to explain the aberrant localization of α-syn in MSA.

### α-Syn overexpression

**Fig. 1:**
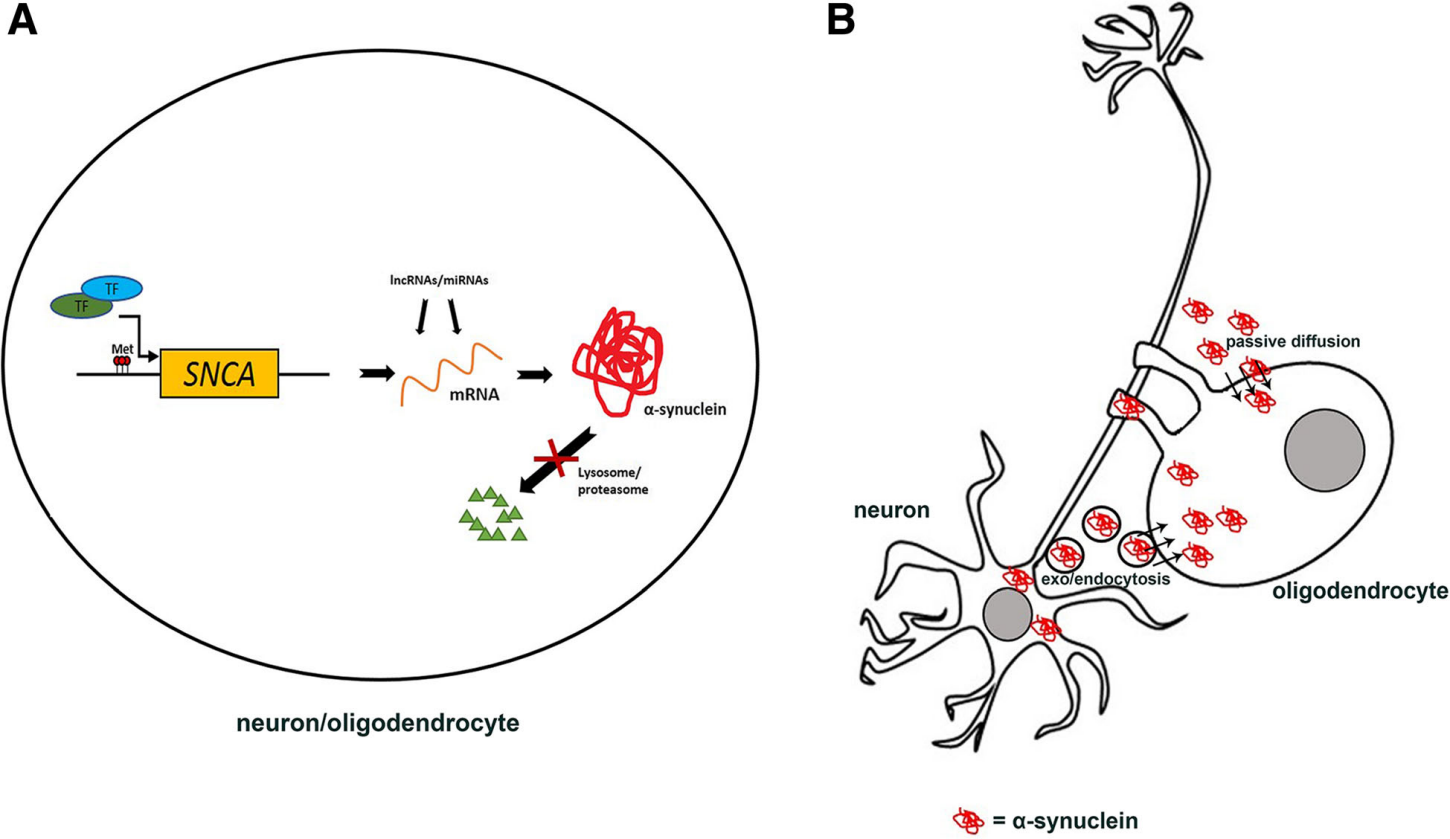
Alpha-synuclein in the pathogenesis of MSA. **a** Putative mechanisms leading to α-syn intracellular accumulation, including *SNCA* CpG islands hypomethylation, transcription factors, lncRNAs, miRNAs, impaired lysosomal and proteasomal machineries. **b** Neuron-oligodendrocyte interaction mechanisms potentially involved in α-syn accumulation: oligodendroglial α-syn uptake from surrounding neurons and extracellular environment through endocytosis and passive transmembrane diffusion. Met = methyl group; TF = transcription factor; miRNAs = microRNAs; lncRNAs = long-non-coding RNAs

The first hypothesis is that a reactivation of α-syn gene (*SNCA*) transcription occurs in the disease (Fig. 1a). The rationale of this conjecture is that an increased transcription would be followed by increased translation and increased protein amount. The putative role of excessive gene expression leading to protein intracellular accumulation is supported by the description of GCIs in oligodendrocytes of brains of PD patients carrying *SNCA* gene triplication [36]. However, in these specific cases, a protein transfer from over-producing neurons is a possible hypothesis. All the studies assessing *SNCA* expression in brains of MSA patients have not found significant differences between MSA and controls [45, 81] or have even detected a downregulation in patients [60]. However, these studies have been performed on RNA extracted from brain samples containing both neurons and glia. Only few studies have investigated the selective expression of *SNCA* in oligodendrocytes of patients and healthy subjects, with conflicting results. In-situ-hybridization analyses on autopsy brain samples [72] have detected a negligible level of *SNCA* mRNA in oligodendrocytes of both patients and controls, thus excluding the possibility that increased *SNCA* transcription may be implicated as cause of the disease. However, two more recent studies [3, 22], based on oligodendrocyte isolation and qPCR analysis, have described a basal gene expression level also in oligodendrocytes, with a trend of increase in MSA patients.

The hypothesis of an aberrant *SNCA* expression in MSA oligodendroglia is intriguing, but the conflicting available data do not allow one to draw definite conclusions about this issue. So far, most of the studies do not support a direct involvement of α-syn gene expression in MSA pathogenesis and the studies suggesting this hypothesis do not provide significant results. However, it must be acknowledged that the isolation of oligodendrocytes from patients’ brains is technically difficult and that the lack of statistical significance may be due to the limited number of subjects used in these studies. Therefore, although *SNCA* overexpression is unlikely to be the sole mechanism leading to the disease onset, it will be crucial to repeat these experiments in wider cohorts of patients and controls, both in brain-isolated and iPSC-derived oligodendrocytes. It will also be important to investigate the role of pre- and post-transcriptional *SNCA* regulatory mechanisms, including CpG island methylation [46, 70], transcription factors [18, 20, 100], lncRNAs [74] and miRNAs [24, 47].

It is interesting to observe that transgenic mice overexpressing human α-syn under the control of promoters of genes specifically expressed in oligodendrocytes (*MBP*, *PLP* and *CNP*) [48, 105, 134], which have represented the gold standard of MSA models for many years, are based on this putative mechanism.

### α-syn uptake from oligodendrocytes

A second hypothesis about the mechanisms leading to α-syn accumulation in MSA suggests that the protein is not produced directly in oligodendroglia, but that it is taken-up from neurons or from the extracellular environment (Fig. 1b). Various studies have demonstrated the ability of neurons to uptake α-syn both in vitro and in vivo [37, 65] and a possible transfer of α-syn from neuron-to-neuron has been demonstrated as well [21]. However, the prominent oligodendroglial pathology in MSA has prompted various laboratories to investigate the possible transfer of α-syn from neurons to oligodendroglia. Oligodendroglial cell lines have shown the ability to uptake α-syn monomers [54, 56] and increased levels of oligodendroglial α-syn have been observed in a double transgenic mouse overexpressing α-syn under *MBP* and *PDGF* promoters, when compared to the *MBP*-mouse model [95]. An extensive study has investigated the uptake of various forms of α-syn from oligodendroglia in vitro and in vivo [92]. Oligodendrocytes are able to internalize α-syn monomers, oligomers and, although to a lesser extent, fibrils. The same species of α-syn can also be internalized in vivo, after injection into the mouse cortex. Moreover, grafted oligodendrocytes can uptake α-syn from host rat neurons overexpressing human α-syn.

All these data are strongly suggestive for a role of the oligodendroglial α-syn uptake mechanism in the pathogenesis of MSA. The precise mechanism by which this happens has not been elucidated yet. However, several studies have investigated putative mechanisms leading to α-syn internalization into neurons, including passive diffusion across cell membrane [2, 62] and endocytosis [37, 62]. This latter mechanism also seems to be implicated in oligodendroglial-mediated uptake, since dynamin inhibition (both genetic and pharmacological) leads to reduced oligodendroglial α-syn uptake and dynamin overexpression enhances α-syn uptake in these cells [54, 92]. A clathrin-dependent internalization mechanism has been suggested as well [56].

### Other α-syn-related hypotheses

A new field of investigation derives from the recent description of a prion-like spreading pathology of α-syn in MSA [51]. The theme of a prion-like α-syn propagation has been widely studied in PD [17]. However, evidence obtained from using brain homogenates/precipitates from MSA patients to inject specific transgenic mice, or in particular cellular assays, has suggested that a unique strain of α-syn prions, different from those observed in PD, may be the causative mechanism of MSA [88, 131–133]. The fact that the in vivo phenotype has been observed only from inoculating MSA samples into Tg M83^+/−^ mice, but not into wildtype mice [88], demands further investigation to better elucidate the issue.

Recent studies have pointed out the role of specific α-syn strains in the pathogenesis of synucleinopathies. For example, it has been shown that α-syn oligomers, ribbons and fibrils exert different effects when injected into rat brains [86]. A recent extensive study [87] has investigated the different conformations and effects of α-syn derived from Lewy bodies (LB-α-syn) or from GCIs (GCI-α-syn), thus providing new hints to understand the specific molecular mechanisms underlying MSA. The authors show that GCI-α-syn and LB-α-syn are characterized by different conformations, as demonstrated by higher resistance of GCI-α-syn to proteinase K digestion and by the different banding patterns obtained after trypsin or thermolysin treatment. Moreover, GCI-α-syn has been shown to be far more potent than LB-α-syn in seeding α-syn aggregation in oligodendrocytes and in inducing neuronal α-syn pathology. Finally, on the basis of various experiments, the authors propose that different cellular subtypes specifically influence the properties of α-syn and that the cellular milieu of oligodendrocytes induces the formation of a particularly aggressive α-syn strain which is different from that obtained when α-syn is incubated in neurons or in neuronal lysates.

Impaired protein degradation may also be involved in α-syn accumulation, as suggested by the description of a possible role of autophagic and proteasomal dysfunction in the disease [16, 75, 76, 103, 112, 117] (Fig. 1a). Furthermore, it has been observed that treating primary rat oligodendrocyte precursor cells with exogenous α-syn-preformed-fibrils increases endogenous α-syn levels through autophagic impairment [50].

It has also been proposed that the accumulation of α-syn may be triggered by specific oligodendroglial proteins, and particular attention has been devoted to p25α/TPPP. Co-expressing α-syn and TPPP in rat oligodendrocytes enhances α-syn aggregation [38]. Furthermore, the relocation of TPPP from myelin sheath to oligodendrocyte’s soma is an early pathological event during the progression of the disease [41].

To sum up, several hypotheses have been proposed to explain the possible origin of aberrant α-syn in MSA, but a definite answer has not been provided yet. At the current time, it cannot be excluded that various mechanisms combine to produce the same final effect.

Another important issue related to α-syn is the putative detrimental effect exerted on the cells in which it accumulates. In this regard, both extrinsic and intrinsic apoptotic pathways may be involved. In particular, the co-expression of α-syn and TPPP in oligodendroglia has been shown to induce the stimulation of Fas receptor and the activation of Caspase 8 [57]. On the other hand, the involvement of the intrinsic apoptotic pathway is suggested by the finding of the mitochondrial pro-apoptotic protein Omi/HtrA2 in GCIs, NCIs and dystrophic neurites [53].

Although the relationship between oligodendroglia and neurons is still a matter of debate and although it has not been elucidated yet whether MSA is a primarily neuronal, oligodendroglial or neuronal-oligodendroglial disease, it is intriguing to hypothesize that α-syn-mediated oligodendroglial pathology at least contributes to neuronal damage, as further supported by the positive correlation between neuronal loss and GCIs density [39, 41, 82]. In this regard, oligodendrocytes play an essential role not only in the formation of the myelin sheath, but also in providing trophic support to neurons. In particular, neuronal survival and axonal length are supported by factors released by oligodendroglial precursors and mature oligodendrocytes. Studies investigating this pathway in MSA have shown that glial cell line-derived neurotrophic factor (GDNF) is reduced in the MBP-h-αsyn-transgenic mice and that the neuropathological and behavioral deficits of these mice are improved by the intracerebroventricular infusion of GDNF [122, 130].

The lack of oligodendroglia-derived neurotrophic factors is not the only mechanism proposed to cause cell-death in the disease [83]. Microglial activation, classically found in MSA brains [41] and probably influenced by α-syn accumulation [11, 116], has been detected also in MSA mouse models. Its association with cell death is supported by the finding of a correlation between microglial activation and dopaminergic neuronal loss, prevented by minocycline-mediated microglial suppression [115]. The finding of an association between microglial activation and the expression of inducible nitric-oxide-synthase (iNOS) [115], whose contribution to neurodegeneration has already been described [30], is also notable.

## Mitochondria

Mitochondria play an important role in several neurodegenerative diseases and, in particular, they have proven to be crucial in the pathogenesis of PD [98]. A defective activity of respiratory chain complex I has been detected in substantia nigra and other tissues of patients affected with PD and the administration of complex I inhibitors (rotenone and MPTP) to animal models and humans has been associated with striatonigral degeneration and parkinsonian features [12, 99, 106]. The finding of increased mtDNA deletions in patients’ brains [9] and the causative role of mutations in mitochondria-related genes (e.g. *Parkin* and *PINK1*) in early-onset PD, are additional clues supporting the role of these organelles in the disease.

**Fig. 2:**
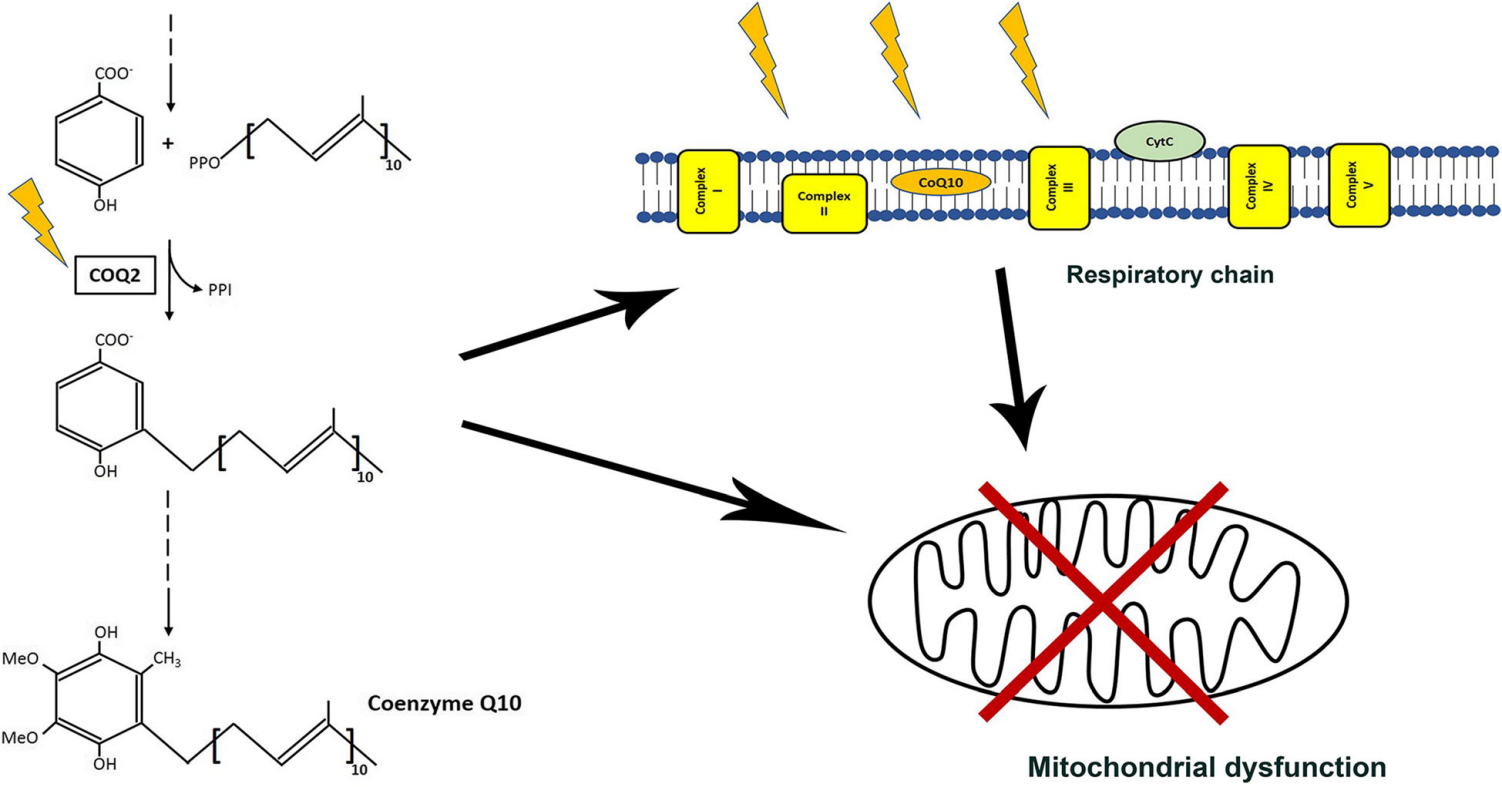
Mitochondria in the pathogenesis of MSA. Figure depicting how specific mitochondrial triggers, including Coenzyme Q10 deficiency and respiratory chain defect, may affect the overall mitochondrial function, thus leading to bioenergetic defect and cellular suffering

Several groups have also investigated the role of mitochondria in MSA (Fig. 2).

Two studies aimed at assessing the activity level of respiratory chain complexes in various tissues of MSA patients and controls have identified reduced complex I activity in patients’ skeletal muscle, but not in substantia nigra or platelets [15, 35]. Moreover, the amount of mitochondrial DNA rearrangements or deletions has not been found to be increased in patients’ substantia nigra [34].

After the recent description [77] of mutations in *COQ2* gene, encoding one of the enzymes involved in Coenzyme Q10 (CoQ10) biosynthesis, in familial and sporadic cases of MSA, the theme of a mitochondrial role in the pathogenesis of the disease has gained new and wider interest. CoQ10, located in the inner mitochondrial membrane, transfers electrons from complexes I and II to complex III, thus playing a crucial role in the functioning of respiratory chain. CoQ10 biosynthesis is a complex biological pathway involving many steps and several enzymes are implicated. Recessive mutations in the genes encoding some of these enzymes, including *COQ2*, [89–91] are responsible for the onset of complex syndromes, often denoted as “primary CoQ10 deficiencies”, which are usually characterized by a prominent neurological dysfunction. After the description of a possible role of *COQ2* mutations in MSA, several groups have sequenced this gene in different patient cohorts. Conflicting results have emerged [63, 79, 96, 102, 104, 135], since some studies, mainly focused on Chinese and Japanese populations, have confirmed the genetic finding while others, mainly focused on Europeans and North Americans, have not.

Since then, the role of CoQ10 in MSA has been further investigated independently from *COQ2* mutations, whose causative effect remains controversial. The evaluation of CoQ10 amount in autopsy brain samples has pointed out a reduction in patients, selectively in the cerebellum and not in other brain areas, including frontal cortex [101], occipital cortex and striatum [5]. The assessment of the activity level of respiratory chain complexes I + III and II + III in brain samples has not provided significant results, whereas the amount of the CoQ10 biosynthesis enzymes PDSS1 and COQ5 has been found to be reduced in patients’ brains. A reduction of CoQ10 level has also been described in patients’ cerebrospinal fluid [19] and plasma/serum [52, 59, 73].

A recent study [75] has investigated several aspects of mitochondrial biology in fibroblasts of MSA patients and controls. An impaired activity of respiratory chain, in particular complex II, and a suboptimal mitophagic machinery have been detected in MSA fibroblasts. The evaluation of CoQ10 pathway has pointed out a reduced CoQ10 amount and an up-regulation of some CoQ10 biosynthesis enzymes (namely COQ5 and COQ7) in patients. Furthermore, analyses on both fibroblasts and peripheral blood cells have suggested an increased mitochondrial content in the cerebellar subtype of the disease.

Mitochondrial functioning has also recently been investigated in dopaminergic neurons differentiated from induced pluripotent stem cells (iPSCs) of 4 MSA patients (2 MSA-P and 2 MSA-C), 4 healthy controls and the healthy monozygotic twin of one of the patients [76]. This study has shown a major involvement of mitochondria in MSA, providing evidence for impaired activity of the respiratory chain (in particular complexes II and II + III), increased amount of respiratory chain complexes II and III, increased mitochondrial mass and up-regulation of CoQ10 biosynthesis, with increased amount of PDSS1, PDSS2, COQ4 and ADCK3/COQ8A. This study not only detected mitochondrial dysfunction in patient neurons, but also neuronal damage and severe impairment of the autophagic machinery.

Mitochondrial dysfunction has been assessed also in another iPSC-based study, investigating iPSC-derived neurons of a patient with a heterozygous mutation in *COQ2* and the corresponding corrected isogenic line, a patient with idiopathic MSA, and three healthy controls. Reduced CoQ10 and vitamin E levels were detected in the *COQ2*-mutated patient. Impaired mitochondrial functioning (assessed by evaluating oxygen consumption rate) was found in both patients. Increased oxidative stress was only found in the *COQ2*-mutated subject, and rescued in the isogenic control. Furthermore, the cells from the patient with COQ2 mutation also displayed increased apoptosis, partially rescued by CoQ10 supplementation [78].

All these studies, observed from a comprehensive point of view, point towards a crucial role of mitochondria in the pathogenesis of MSA. It is still not clear whether mitochondrial defects represent the primary cause of the disease or a secondary effect. However, it is plausible to hypothesize that, once mitochondrial impairment occurs, this contributes to bioenergetics dysfunction, cellular damage, and ultimately neurodegeneration.

## Other mechanisms involved in MSA pathogenesis

Although the present review is specifically focused on α-syn accumulation and mitochondrial dysfunction, it must be acknowledged that other hypotheses have been proposed to explain the pathogenesis of MSA.

As previously stated, the role of inflammation has been extensively investigated in the disease and it is important to highlight the most significant findings in this field. Microglial activation is commonly detectable in patients’ brains [40, 41] and, although the underlying mechanism is not completely clear, in vivo and in vitro analyses have shown that α-syn may be involved in this process [11, 116]. However, oxidative stress is thought to play an important role as well [114]. As already mentioned, the microglial activation which can be detected in MSA mouse models correlates with neuronal loss in the substantia nigra and the observed increased iNOS expression may contribute to this effect [115]. Toll-like-receptor 4, also found to be up-regulated in these mice [115], is proposed to play a protective role because its ablation in PLP-transgenic-mice leads to clinical and neuropathological worsening and upregulation of various inflammatory mediators [110]. Furthermore, it has been shown that inhibiting myeloperoxidase, an enzyme implicated in reactive oxygen species production, in MSA mouse models leads to an improvement of clinical and neuropathological features and reduces microglial activation [111]. Overall, several pieces of evidence are strongly suggestive for an important role of inflammation in MSA. Although the activation of the inflammatory cascade may be secondary to other phenomena, including α-syn accumulation and mitochondrial dysfunction, it likely contributes to many of the detrimental processes which can be observed in the disease.

The role of impaired protein degradation has to also be considered when discussing the pathogenic mechanisms of MSA. Neuropathological studies of MSA brains suggest an involvement of autophagy in the disease, as supported, for example, by the description of GCIs’ positive staining for LC3 [103, 117]. The finding of an up-regulation of microRNA-101 in the striatum of MSA patients and the demonstration that overexpression of this molecule in cell cultures is accompanied by autophagic impairment and increased α-syn has led to the hypothesis that microRNA-101 dysregulation may contribute to MSA pathogenesis by altering the autophagic pathway [125]. A recent iPSC-based study, already mentioned when discussing mitochondrial dysfunction, has shown that MSA neurons are characterized by a severe autophagic impairment, as demonstrated by increased basal autophagy, reduced autophagic flux and reduced activity of the lysosomal enzymes α-Mannosidase and β-Mannosidase [76]. Finally, in addition to the classical autophagy-mediated intracellular degradation system, the proteasomal machinery may also be affected. Systemic proteasome inhibition has been shown to worsen clinical and neuropathological features in PLP-transgenic-mice, but not in wild-type mice [112] and proteasomal structural abnormalities have been observed in patients’ substantia nigra [16].

It has also been proposed that alterations in lipid metabolism and myelin formation may be involved in MSA pathogenesis. This hypothesis is supported by the finding of altered sphingomyelin, sulfatide and galactosylceramide in affected white matter of MSA brains [23]. Myelin defects have been correlated with an altered expression of ATP-binding cassette transporter A8, which may be involved in myelin formation and maintenance [13, 14]. Furthermore, various studies have shown that α-syn accumulation negatively affects the maturation of oligodendrocyte precursor cells and the myelination process [25, 26, 71].

The finding of an increased iron level in specific brain regions of MSA patients has led to the hypothesis that iron metabolism dysregulation may play a role in the pathogenesis of the disease. Although the issue remains very unclear, iron metabolism dysregulation, inflammation and α-syn accumulation may be closely related. Iron is thought to induce oxidative stress and to activate microglia, thus fostering the inflammatory process. These phenomena have been proposed to influence α-syn-related pathology by inducing α-syn aggregation, post-translational modifications and conformational changes [49].

## Conclusions

Although several efforts have been dedicated to unravelling the causes of MSA, the precise pathogenic mechanisms underlying this disorder still have to be elucidated.

The peculiar neuropathological pattern of the disease, characterized by α-syn accumulation in oligodendrocytes, has led many investigators to focus on this particular aspect and to hypothesize that MSA primarily represents an oligodendrogliopathy, with a secondary neuronal involvement. However, several hints, mainly emerged from recent iPSC-based studies, have shown that a pathological phenotype can be observed also in neurons, independently from oligodendrocytes. In this perspective, although the findings are still preliminary, it is reasonable to hypothesize that both neurons and oligodendrocytes may be primarily affected and that the damage of one cell type contributes to the degeneration of the other, and viceversa. Therefore, the expression “oligodendroglioneural synucleinopathy”, recently proposed to describe MSA [42], may properly suit the disease.

The temporal sequence of pathogenic events is still obscure and it is not clear which of the proposed causative mechanisms (e.g. protein accumulation, mitochondrial dysfunction, inflammation) represents the primary episode which triggers the whole pathogenic cascade.

The peculiar oligodendroglial pathological presentation of MSA has induced many investigators to hypothesize that the primitive cause of the disease has to be related to α-syn accumulation in this cellular subtype and this is also the rationale underlying MSA transgenic mouse models. These mice, which overexpress human α-syn in oligodendrocytes, are the main supporting evidence for the “α-syn-primary-hit” hypothesis of MSA pathogenesis. Indeed, they are characterized by several clinical and neuropathological features, including secondary neurodegeneration.

**Fig. 3:**
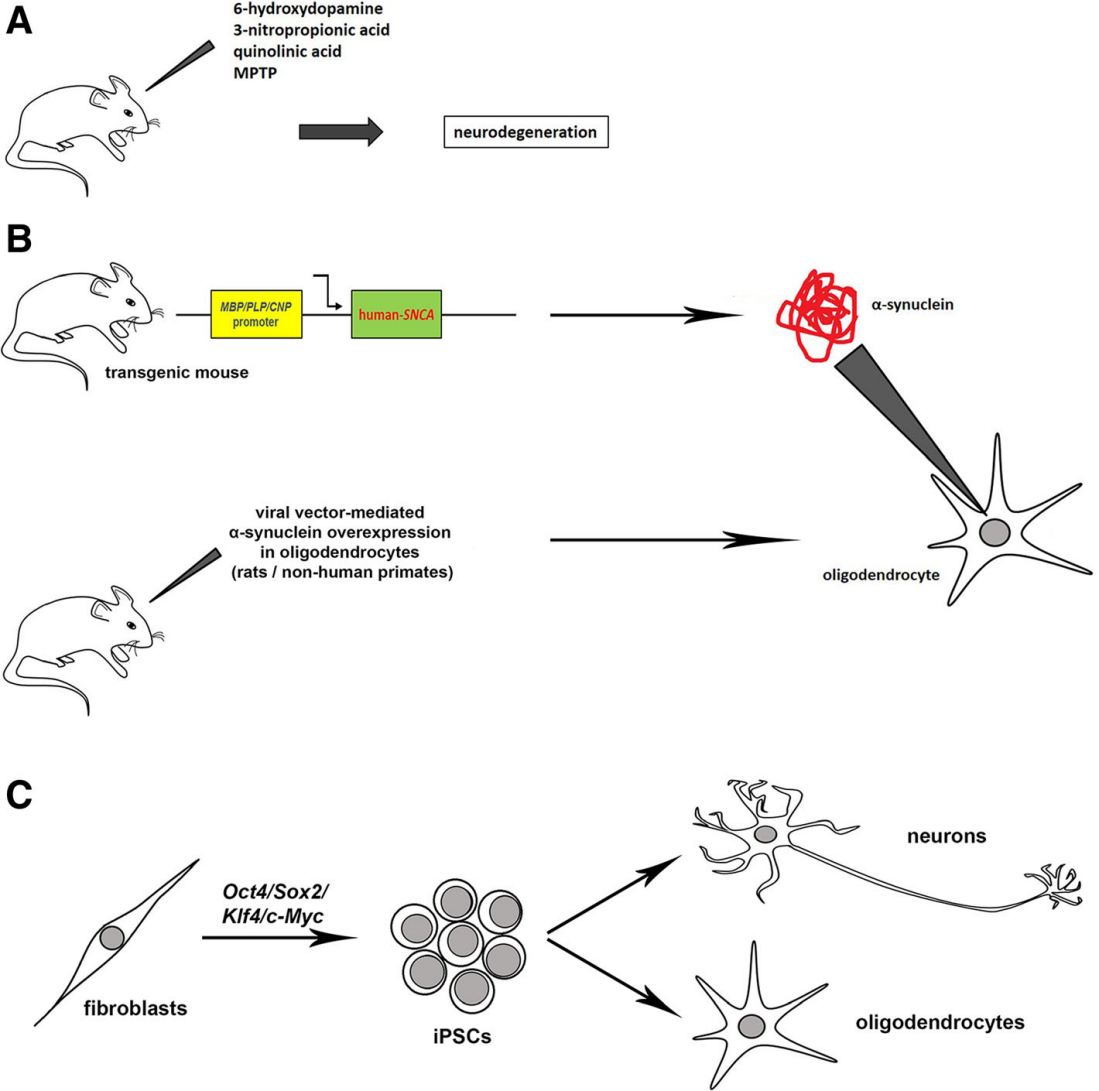
Available models of MSA. **a** Toxin-induced models of MSA. The first models of MSA have been produced by injecting specific toxins (e.g. 6-hydroxydopamine, 3-nitropropionic acid, quinolinic acid and MPTP) into animals, thus inducing neurodegeneration of specific brain areas. These models are no longer considered as relevant disease models and have been substantially abandoned. **b** Animal models of MSA, obtained overexpressing human α-syn specifically in oligodendrocytes. The upper part of the panel shows transgenic mice which overexpress h-*SNCA* under the control of promoters of genes expressed in oligodendrocytes, namely myelin basic protein (*MBP*), proteolipid protein (*PLP*) or 2′,3′-cyclic nucleotide 3′-phosphodiesterase (*CNP*). The lower part of the panel shows a recently developed model obtained overexpressing human α-syn in oligodendrocytes of rodents and primates through viral vectors. **c** iPSC-based models of MSA. The expression of specific factors (*Oct4*, *Sox2*, *Klf4* and *c-Myc*) allows to reprogram adult somatic cells, including fibroblasts and leukocytes, to induced pluripotent stem cells, which can then be differentiated toward all the different cellular subtypes of the organism, including neurons and glia

On the other hand, recent studies are supportive for a causative role of mitochondria in the pathogenesis of MSA. The finding of putatively causative mutations in *COQ2* gene in familial and sporadic cases of MSA, although still controversial, would represent, if definitely confirmed, the most direct evidence. The finding of mitochondrial dysfunction in patients’ fibroblasts and iPSC-derived neurons independently from α-syn accumulation is a further piece of evidence supporting this hypothesis [75, 76]. It is also interesting to highlight that one of the first in vivo models of the disease (not used anymore) has been obtained by administering the succinate dehydrogenase (respiratory chain complex II) inhibitor 3-nitroproprionic acid (3-NP acid) to animals (Fig. 3a) [29, 113, 120] for its ability to cause a striatal lesion. It is noteworthy that complex II deficiency has been recently observed in MSA cellular models [75, 76] and the relationship between complex II defect and striatal involvement is worth of further investigation. Finally, at least one of the alternative proposed pathogenic mechanisms, inflammation, may be easily related to mitochondrial dysfunction, which notoriously leads to increased oxidative stress and, subsequently, inflammation [64].

Another possibility is that both α-syn accumulation and mitochondrial dysfunction are necessary for the onset of the disease, thus playing a synergic effect. This hypothesis is supported by studies performed on transgenic mice overexpressing α-syn in oligodendrocytes and treated with the mitochondrial toxin 3-NP acid [114, 120], which are characterized by worsening of both clinical and neuropathological outcomes compared to untreated animals. Furthermore, a possible relationship between α-syn accumulation and mitochondrial dysfunction has already been described [94].

Therefore, it is intriguing to hypothesize that MSA represents a multifactorial disease caused by the combined effect of multiple hits. It is also possible that genetic, epigenetic and environmental risk factors play a synergic role, eventually leading to the disease onset. However, although this hypothesis is suggestive and would partially explain the difficulty in finding a univocal mechanism, further investigation is needed.

In the perspective of intensifying the efforts devoted at understanding these mechanisms, two main issues should be addressed.

First, wide studies aimed at identifying new genetic hallmarks of the disease, both inherited in classical mendelian fashion or just playing the role of risk factors, should be encouraged. Epigenetic factors may also play a role and specific studies should address this specific topic. In this perspective, the availability of couples of monozygotic twins discordant for the disease, already described in previous studies [76], may be useful.

Second, the generation of new models will be crucial. Transgenic mice have represented the gold standard of MSA models for many years and have allowed to dissect several important aspects of the disease. However, their main limit is that α-syn is artificially overexpressed in oligodendrocytes, without explaining the mechanism which leads to the accumulation itself and underestimating the role of other cells, including neurons. (Fig. 3b) Although these mice are still necessary for several reasons, including the necessity to test novel therapeutic compounds in vivo, there is an increasing need of novelties in this field. The viral vector-mediated overexpression of alpha-synuclein in oligodendrocytes of rats and non-human primates, described in two recent papers [8, 66], is based on the same rationale of transgenic mice, but the different methodological and technical approach may provide interesting and unexpected outcomes. (Fig. 3b) Another cutting-edge technology which has recently been used to model MSA is represented by iPSC-derived neurons and glia. This technique, based on a completely different approach, allows to obtain a human patient-specific model of the disease. (Fig. 3c) Although the description of familial MSA cases is very rare and although iPSC-based technology is optimal for diseases with a defined genetic cause, recent studies have shown that this technique may be useful to model idiopathic cases of this supposedly sporadic disorder [22, 76, 78].

The importance of understanding the molecular mechanisms of the disease has not only purely speculative purposes, but also finds practical applications in identifying new biomarkers and therapeutic approaches. The lack of effective therapies for MSA further urges basic and translational research in this field. Although most of the proposed pathogenic mechanisms do not find a clinical application yet, some pre-clinical and clinical trials are ongoing. The field which has raised more interest so far is represented by pharmacological compounds which target α-syn: in this perspective, it has been proposed to act at different levels, including α-syn expression, α-syn aggregation, α-syn degradation and clearance and α-syn cell-to-cell propagation [123]. A notable example is represented by α-syn immunotherapy (both passive and active immunization), which has shown promising results in preclinical models of synucleinopathies [4, 31, 67–69, 118] and is now under investigation in clinical trials. However, other pharmacological compounds which are not directly related to α-syn, but to independent pathogenic mechanisms, including inflammation [111, 124] and mitochondrial dysfunction [59], are under investigation.

To conclude, a remarkable amount of work has already been done to unravel the pathogenesis of MSA and several dysfunctional pathways have been detected. However, the lack of a definite mechanism demands further and more intense efforts. The identification of new therapeutic targets for this still incurable disease will largely depend on the identification of its molecular causes.

## Abbreviations

CNP: 2′,3′-cyclic nucleotide 3′-phosphodiesterase
CoQ10: Coenzyme Q10
GCIs: glial cytoplasmic inclusions
GDNF: glial cell line-derived neurotrophic factor
iNOS: inducible nitric-oxide-synthase
iPSCs: induced pluripotent stem cells
LB: Lewy bodies
lncRNA: long-non-coding RNA
MBP: myelin basic protein
miRNA: microRNA
MPTP: 1-methyl-4-phenyl-1,2,3,6-tetrahydropyridine
MSA: Multiple System Atrophy
NCIs: neuronal cytoplasmic inclusions
PD: Parkinson’s disease
PDGF: platelet-derived growth factor
PLP: proteolipid protein
α-syn: α-synuclein

## References

[1] Ahmed Z, Asi YT, Lees AJ, Revesz T, Holton JL (2013). Identification and quantification of oligodendrocyte precursor cells in multiple system atrophy, progressive supranuclear palsy and Parkinson’s disease. Brain Pathol.

[2] Ahn KJ, Paik SR, Chung KC, Kim J (2006). Amino acid sequence motifs and mechanistic features of the membrane translocation of alpha-synuclein. J Neurochem.

[3] Asi YT, Simpson JE, Heath PR, Wharton SB, Lees AJ, Revesz T (2014). Alpha-synuclein mRNA expression in oligodendrocytes in MSA. Glia.

[4] Bae EJ, Lee HJ, Rockenstein E, Ho DH, Park EB, Yang NY (2012). Antibody-aided clearance of extracellular α-synuclein prevents cell-to-cell aggregate transmission. J Neurosci.

[5] Barca E, Kleiner G, Tang G, Ziosi M, Tadesse S, Masliah E (2016). Decreased coenzyme Q10 levels in multiple system atrophy cerebellum. J Neuropathol Exp Neurol.

[6] Barrett PJ, Timothy Greenamyre J (2015). Post-translational modification of α-synuclein in Parkinson's disease. Brain Res.

[7] Bartels T, Choi JG, Selkoe DJ (2011). α-Synuclein occurs physiologically as a helically folded tetramer that resists aggregation. Nature.

[8] Bassil F, Guerin PA, Dutheil N, Li Q, Klugmann M, Meissner WG (2017). Viral-mediated oligodendroglial alpha-synuclein expression models multiple system atrophy. Mov Disord.

[9] Bender A, Krishnan KJ, Morris CM, Taylor GA, Reeve AK, Perry RH (2006). High levels of mitochondrial DNA deletions in substantia nigra neurons in aging and Parkinson disease. Nat Genet.

[10] Bendor JT, Logan TP, Edwards RH (2013). The function of α-synuclein. Neuron.

[11] Béraud D, Hathaway HA, Trecki J, Chasovskikh S, Johnson DA, Johnson JA (2013). Microglial activation and antioxidant responses induced by the Parkinson’s disease protein α-synuclein. J NeuroImmune Pharmacol.

[12] Betarbet R, Sherer TB, MacKenzie G, Garcia-Osuna M, Panov AV, Greenamyre JT (2000). Chronic systemic pesticide exposure reproduces features of Parkinson's disease. Nat Neurosci.

[13] Bleasel JM, Hsiao JH, Halliday GM, Kim WS (2013). Increased expression of ABCA8 in multiple system atrophy brain is associated with changes in pathogenic proteins. Parkinsons Dis.

[14] Bleasel JM, Wong JH, Halliday GM, Kim WS (2014). Lipid dysfunction and pathogenesis of multiple system atrophy. Acta Neuropathol Commun.

[15] Blin O, Desnuelle C, Rascol O, Borg M, Peyro Saint Paul H, Azulay JP (1994). Mitochondrial respiratory failure in skeletal muscle from patients with Parkinson’s disease and multiple system atrophy. J Neurol Sci.

[16] Bukhatwa S, Zeng BY, Rose S, Jenner P (2010). A comparison of changes in proteasomal subunit expression in the substantia nigra in Parkinson's disease, multiple system atrophy and progressive supranuclear palsy. Brain Res.

[17] Chu Y, Kordower JH (2015). The prion hypothesis of Parkinson's disease. Curr Neurol Neurosci Rep.

[18] Clough RL, Dermentzaki G, Stefanis L (2009). Functional dissection of the alpha-synuclein promoter: transcriptional regulation by ZSCAN21 and ZNF219. J Neurochem.

[19] Compta Y, Giraldo DM, Muñoz E, Antonelli F, Fernández M, Bravo P (2018). Cerebrospinal fluid levels of coenzyme Q10 are reduced in multiple system atrophy. Parkinsonism Relat Disord.

[20] Dermentzaki G, Paschalidis N, Politis PK, Stefanis L (2016). Complex effects of the ZSCAN21 transcription factor on transcriptional regulation of α-Synuclein in primary neuronal cultures and in vivo. J Biol Chem.

[21] Desplats P, Lee HJ, Bae EJ, Patrick C, Rockenstein E, Crews L (2009). Inclusion formation and neuronal cell death through neuron-to-neuron transmission of alpha-synuclein. Proc Natl Acad Sci U S A.

[22] Djelloul M, Holmqvist S, Boza-Serrano A, Azevedo C, Yeung MS, Goldwurm S (2015). Alpha-Synuclein expression in the oligodendrocyte lineage: an in vitro and in vivo study using rodent and human models. Stem Cell Rep.

[23] Don AS, Hsiao JH, Bleasel JM, Couttas TA, Halliday GM, Kim WS (2014). Altered lipid levels provide evidence for myelin dysfunction in multiple system atrophy. Acta Neuropathol Commun.

[24] Doxakis E (2010). Post-transcriptional regulation of alpha-synuclein expression by mir-7 and mir-153. J Biol Chem.

[25] Ettle B, Kerman BE, Valera E, Gillmann C, Schlachetzki JC, Reiprich S (2016). α-Synuclein-induced myelination deficit defines a novel interventional target for multiple system atrophy. Acta Neuropathol.

[26] Ettle B, Reiprich S, Deusser J, Schlachetzki JC, Xiang W, Prots I (2014). Intracellular alpha-synuclein affects early maturation of primary oligodendrocyte progenitor cells. Mol Cell Neurosci.

[27] Fanciulli A, Wenning GK (2015). Multiple-system atrophy. N Engl J Med.

[28] Fauvet B, Mbefo MK, Fares MB, Desobry C, Michael S, Ardah MT (2012). α-Synuclein in central nervous system and from erythrocytes, mammalian cells, and Escherichia coli exists predominantly as disordered monomer. J Biol Chem.

[29] Fernagut PO, Diguet E, Stefanova N, Biran M, Wenning GK, Canioni P (2002). Subacute systemic 3-nitropropionic acid intoxication induces a distinct motor disorder in adult C57Bl/6 mice: behavioural and histopathological characterisation. Neuroscience.

[30] Förstermann U, Sessa WC (2012). Nitric oxide synthases: regulation and function. Eur Heart J.

[31] Games D, Valera E, Spencer B, Rockenstein E, Mante M, Adame A (2014). Reducing C-terminal-truncated alpha-synuclein by immunotherapy attenuates neurodegeneration and propagation in Parkinson's disease-like models. J Neurosci.

[32] Gilman S, May SJ, Shults CW, Tanner CM, Kukull W, Lee VM (2005). The north American multiple system atrophy study group. J Neural Transm (Vienna).

[33] Gilman S, Wenning GK, Low PA, Brooks DJ, Mathias CJ, Trojanowski JQ (2008). Second consensus statement on the diagnosis of multiple system atrophy. Neurology.

[34] Gu G, Reyes PE, Golden GT, Woltjer RL, Hulette C, Montine TJ, Zhang J (2002). Mitochondrial DNA deletions/rearrangements in parkinson disease and related neurodegenerative disorders. J Neuropathol Exp Neurol.

[35] Gu M, Gash MT, Cooper JM, Wenning GK, Daniel SE, Quinn NP (1997). Mitochondrial respiratory chain function in multiple system atrophy. Mov Disord.

[36] Gwinn-Hardy K, Mehta ND, Farrer M, Maraganore D, Muenter M, Yen SH (2000). Distinctive neuropathology revealed by alpha-synuclein antibodies in hereditary parkinsonism and dementia linked to chromosome 4p. Acta Neuropathol.

[37] Hansen C, Angot E, Bergström AL, Steiner JA, Pieri L, Paul G (2011). α-Synuclein propagates from mouse brain to grafted dopaminergic neurons and seeds aggregation in cultured human cells. J Clin Invest.

[38] Hasegawa T, Baba T, Kobayashi M, Konno M, Sugeno N, Kikuchi A (2010). Role of TPPP/p25 on α-synuclein-mediated oligodendroglial degeneration and the protective effect of SIRT2 inhibition in a cellular model of multiple system atrophy. Neurochem Int.

[39] Inoue M, Yagishita S, Ryo M, Hasegawa K, Amano N, Matsushita M (1997). The distribution and dynamic density of oligodendroglial cytoplasmic inclusions (GCIs) in multiple system atrophy: a correlation between the density of GCIs and the degree of involvement of striatonigral and olivopontocerebellar systems. Acta Neuropathol.

[40] Ishizawa K, Komori T, Sasaki S, Arai N, Mizutani T, Hirose T (2004). Microglial activation parallels system degeneration in multiple system atrophy. J Neuropathol Exp Neurol.

[41] Jellinger KA (2014). Neuropathology of multiple system atrophy: new thoughts about pathogenesis. Mov Disord.

[42] Jellinger KA (2018). Multiple system atrophy: An Oligodendroglioneural Synucleinopathy. J Alzheimers Dis.

[43] Jellinger KA, Lantos PL (2010). Papp-Lantos inclusions and the pathogenesis of multiple system atrophy: an update. Acta Neuropathol.

[44] Jellinger KA, Wenning GK (2016). Multiple system atrophy: pathogenic mechanisms and biomarkers. J Neural Transm (Vienna).

[45] Jin H, Ishikawa K, Tsunemi T, Ishiguro T, Amino T, Mizusawa H (2008). Analyses of copy number and mRNA expression level of the alpha-synuc lein gene in multiple system atrophy. J Med Dent Sci.

[46] Jowaed A, Schmitt I, Kaut O, Wüllner U (2010). Methylation regulates alpha-synuclein expression and is decreased in Parkinson's disease patients’ brains. J Neurosci.

[47] Junn E, Lee KW, Jeong BS, Chan TW, Im JY, Mouradian MM (2009). Repression of alpha-synuclein expression and toxicity by microRNA-7. Proc Natl Acad Sci U S A.

[48] Kahle PJ, Neumann M, Ozmen L, Muller V, Jacobsen H, Spooren W (2002). Hyperphosphorylation and insolubility of α-synuclein in transgenic mouse oligodendrocytes. EMBO Rep.

[49] Kaindlstorfer C, Jellinger KA, Eschlböck S, Stefanova N, Weiss G, Wenning GK (2018). The relevance of Iron in the pathogenesis of multiple system atrophy: a viewpoint. J Alzheimers Dis.

[50] Kaji S, Maki T, Kinoshita H, Uemura N, Ayaki T, Kawamoto Y (2018). Pathological endogenous α-Synuclein accumulation in oligodendrocyte precursor cells potentially induces inclusions in multiple system atrophy. Stem Cell Rep.

[51] Karpowicz Richard J., Trojanowski John Q., Lee Virginia M.-Y. (2019). Transmission of α-synuclein seeds in neurodegenerative disease: recent developments. Laboratory Investigation.

[52] Kasai T, Tokuda T, Ohmichi T, Ishii R, Tatebe H, Nakagawa M, Mizuno T (2016). Serum levels of coenzyme Q10 in patients with multiple system atrophy. PLoS One.

[53] Kawamoto Y, Kobayashi Y, Suzuki Y, Inoue H, Tomimoto H, Akiguchi I (2008). Accumulation of HtrA2/Omi in neuronal and glial inclusions in brains with alpha-synucleinopathies. J Neuropathol Exp Neurol.

[54] Kisos H, Pukaß K, Ben-Hur T, Richter-Landsberg C, Sharon R (2012). Increased neuronal α-synuclein pathology associates with its accumulation in oligodendrocytes in mice modeling α-synucleinopathies. PLoS One.

[55] Köllensperger M, Geser F, Ndayisaba JP, Boesch S, Seppi K, Ostergaard K (2010). Presentation, diagnosis, and management of multiple system atrophy in Europe: final analysis of the European multiple system atrophy registry. Mov Disord.

[56] Konno M, Hasegawa T, Baba T, Miura E, Sugeno N, Kikuchi A (2012). Suppression of dynamin GTPase decreases α-synuclein uptake by neuronal and oligodendroglial cells: a potent therapeutic target for synucleinopathy. Mol Neurodegener.

[57] Kragh CL, Fillon G, Gysbers A, Hansen DH, Neumann M, Richter-Landsberg C (2013). FAS-dependent cell death in α-synuclein transgenic oligodendrocyte models of multiple system atrophy. PLoS One.

[58] Krismer F, Wenning GK (2017). Multiple system atrophy: insights into a rare and debilitating movement disorder. Nat Rev Neurol.

[59] Kuo SH, Quinzii MC (2016). Coenzyme Q10 as a peripheral biomarker for multiple system atrophy. JAMA Neurol.

[60] Langerveld AJ, Mihalko D, DeLong C, Walburn J, Ide CF (2007). Gene expression changes in postmortem tissue from the rostral pons of multiple system atrophy patients. Mov Disord.

[61] Lashuel HA, Overk CR, Oueslati A, Masliah E (2013). The many faces of α-synuclein: from structure and toxicity to therapeutic target. Nat Rev Neurosci.

[62] Lee HJ, Suk JE, Bae EJ, Lee JH, Paik SR, Lee SJ (2008). Assembly-dependent endocytosis and clearance of extracellular alpha-synuclein. Int J Biochem Cell Biol.

[63] Lin CH, Tan EK, Yang CC, Yi Z, Wu RM (2015). COQ2 gene variants associate with cerebellar subtype of multiple system atrophy in Chinese. Mov Disord.

[64] Lugrin J, Rosenblatt-Velin N, Parapanov R, Liaudet L (2014). The role of oxidative stress during inflammatory processes. Biol Chem.

[65] Luk KC, Kehm VM, Zhang B, O'Brien P, Trojanowski JQ, Lee VM (2012). Intracerebral inoculation of pathological α-synuclein initiates a rapidly progressive neurodegenerative α-synucleinopathy in mice. J Exp Med.

[66] Mandel RJ, Marmion DJ, Kirik D, Chu Y, Heindel C, McCown T (2017). Novel oligodendroglial alpha synuclein viral vector models of multiple system atrophy: studies in rodents and nonhuman primates. Acta Neuropathol Commun.

[67] Mandler M, Valera E, Rockenstein E, Mante M, Weninger H, Patrick C (2015). Active immunization against alpha-synuclein ameliorates the degenerative pathology and prevents demyelination in a model of multiple system atrophy. Mol Neurodegener.

[68] Mandler M, Valera E, Rockenstein E, Weninger H, Patrick C, Adame A (2014). Next-generation active immunization approach for synucleinopathies: implications for Parkinson’s disease clinical trials. Acta Neuropathol.

[69] Masliah E, Rockenstein E, Mante M, Crews L, Spencer B, Adame A (2011). Passive immunization reduces behavioral and neuropathological deficits in an alpha-synuclein transgenic model of Lewy body disease. PLoS One.

[70] Matsumoto L, Takuma H, Tamaoka A, Kurisaki H, Date H, Tsuji S, Iwata A (2010). CpG demethylation enhances alpha-synuclein expression and affects the pathogenesis of Parkinson's disease. PLoS One.

[71] May VE, Ettle B, Poehler AM, Nuber S, Ubhi K, Rockenstein E (2014). α-Synuclein impairs oligodendrocyte progenitor maturation in multiple system atrophy. Neurobiol Aging.

[72] Miller DW, Johnson JM, Solano SM, Hollingsworth ZR, Standaert DG, Young AB (2005). Absence of alpha-synuclein mRNA expression in normal and multiple system atrophy oligodendroglia. J Neural Transm (Vienna).

[73] Mitsui J, Matsukawa T, Yasuda T, Ishiura H, Tsuji S (2016). Plasma coenzyme Q10 levels in patients with multiple system atrophy. JAMA Neurol.

[74] Mizuta I, Takafuji K, Ando Y, Satake W, Kanagawa M, Kobayashi K (2013). YY1 binds to α-synuclein 3′-flanking region SNP and stimulates antisense noncoding RNA expression. J Hum Genet.

[75] Monzio Compagnoni G, Kleiner G, Bordoni A, Fortunato F, Ronchi D, Salani S (2018). Mitochondrial dysfunction in fibroblasts of multiple system atrophy. Biochim Biophys Acta Mol basis Dis.

[76] Monzio Compagnoni G, Kleiner G, Samarani M, Aureli M, Faustini G, Bellucci A (2018). Mitochondrial dysregulation and impaired autophagy in iPSC-derived dopaminergic neurons of multiple system atrophy. Stem Cell Rep.

[77] Multiple-System Atrophy Research Collaboration (2013). Mutations in COQ2 in familial and sporadic multiple-system atrophy. N Engl J Med.

[78] Nakamoto FK, Okamoto S, Mitsui J, Sone T, Ishikawa M, Yamamoto Y (2018). The pathogenesis linked to coenzyme Q10 insufficiency in iPSC-derived neurons from patients with multiple-system atrophy. Sci Rep.

[79] Ogaki K, Fujioka S, Heckman MG, Rayaprolu S, Soto-Ortolaza AI, Labbé C (2014). Analysis of COQ2 gene in multiple system atrophy. Mol Neurodegener.

[80] Oueslati A, Fournier M, Lashuel HA (2010). Role of post-translational modifications in modulating the structure, function and toxicity of alpha-synuclein: implications for Parkinson's disease pathogenesis and therapies. Prog Brain Res.

[81] Ozawa T, Okuizumi K, Ikeuchi T, Wakabayashi K, Takahashi H, Tsuji S (2001). Analysis of the expression level of alpha-synuclein mRNA using postmortem brain samples from pathologically confirmed cases of multiple system atrophy. Acta Neuropathol.

[82] Ozawa T, Paviour D, Quinn NP, Josephs KA, Sangha H, Kilford L (2004). The spectrum of pathological involvement of the striatonigral and olivopontocerebellar systems in multiple system atrophy: clinicopathological correlations. Brain.

[83] Palma JA, Kaufmann H (2015). Novel therapeutic approaches in multiple system atrophy. Clin Auton Res.

[84] Papp MI, Kahn JE, Lantos PL (1989). Glial cytoplasmic inclusions in the CNS of patients with multiple system atrophy (striatonigral degeneration, olivopontocerebellar atrophy and shy-Drager syndrome). J Neurol Sci.

[85] Papp MI, Lantos PL (1994). The distribution of oligodendroglial inclusions in multiple system atrophy and its relevance to clinical symptomatology. Brain.

[86] Peelaerts W, Bousset L, Van der Perren A, Moskalyuk A, Pulizzi R, Giugliano M (2015). α-Synuclein strains cause distinct synucleinopathies after local and systemic administration. Nature.

[87] Peng C, Gathagan RJ, Covell DJ, Medellin C, Stieber A, Robinson JL (2018). Cellular milieu imparts distinct pathological α-synuclein strains in α-synucleinopathies. Nature.

[88] Prusiner SB, Woerman AL, Mordes DA, Watts JC, Rampersaud R, Berry DB (2015). Evidence for α-synuclein prions causing multiple system atrophy in humans with parkinsonism. Proc Natl Acad Sci U S A.

[89] Quinzii C, Naini A, Salviati L, Trevisson E, Navas P, Dimauro S, Hirano M (2006). A mutation in Para-hydroxybenzoate-polyprenyl transferase (COQ2) causes primary coenzyme Q10 deficiency. Am J Hum Genet.

[90] Quinzii CM, Hirano M (2011). Primary and secondary CoQ(10) deficiencies in humans. Biofactors.

[91] Quinzii CM, Hirano M, DiMauro S (2014). Mutant COQ2 in multiple-system atrophy. N Engl J Med.

[92] Reyes JF, Rey NL, Bousset L, Melki R, Brundin P, Angot E (2014). Alpha-synuclein transfers from neurons to oligodendrocytes. Glia.

[93] Richter-Landsberg C, Gorath M, Trojanowski JQ, Lee VM (2000). Alpha-synuclein is developmentally expressed in cultured rat brain oligodendrocytes. J Neurosci Res.

[94] Rocha EM, De Miranda B, Sanders LH (2018). Alpha-synuclein: pathology, mitochondrial dysfunction and neuroinflammation in Parkinson's disease. Neurobiol Dis.

[95] Rockenstein E, Ubhi K, Inglis C, Mante M, Patrick C, Adame A, Masliah E (2012). Neuronal to oligodendroglial alpha-synuclein redistribution in a double transgenic model of MSA. Neuroreport.

[96] Ronchi D, Di Biase E, Franco G, Melzi V, Del Sorbo F, Elia A (2016). Mutational analysis of COQ2 in patients with MSA in Italy. Neurobiol Aging.

[97] Sato K, Kaji R, Matsumoto S, Goto S (2007). Cell type-specific neuronal loss in the putamen of patients with multiple system atrophy. Mov Disord.

[98] Schapira AH (2008). Mitochondria in the aetiology and pathogenesis of Parkinson's disease. Lancet Neurol.

[99] Schapira AH, Cooper JM, Dexter D, Jenner P, Clark JB, Marsden CD (1989). Mitochondrial complex I deficiency in Parkinson's disease. Lancet.

[100] Scherzer CR, Grass JA, Liao Z, Pepivani I, Zheng B, Eklund AC (2008). GATA transcription factors directly regulate the Parkinson's disease-linked gene alpha-synuclein. Proc Natl Acad Sci U S A.

[101] Schottlaender LV, Bettencourt C, Kiely AP, Chalasani A, Neergheen V, Holton JL (2016). Coenzyme Q10 levels are decreased in the cerebellum of multiple-system atrophy patients. PLoS One.

[102] Schottlaender LV, Houlden H, Multiple-System Atrophy (MSA) Brain Bank Collaboration (2014). Mutant COQ2 in multiple-system atrophy. N Engl J Med.

[103] Schwarz L, Goldbaum O, Bergmann M, Probst-Cousin S, Richter-Landsberg C (2012). Involvement of macroautophagy in multiple system atrophy and protein aggregate formation in oligodendrocytes. J Mol Neurosci.

[104] Sharma M, Wenning G, Krüger R, European multiple-system atrophy study group (EMSA-SG) (2014). Mutant COQ2 in multiple-system atrophy. N Engl J Med.

[105] Shults CW, Rockenstein E, Crews L, Adame A, Mante M, Larrea G (2005). Neurological and neurodegenerative alterations in a transgenic mouse model expressing human alpha-synuclein under oligodendrocyte promoter: implications for multiple system atrophy. J Neurosci.

[106] Singer TP, Salach JI, Castagnoli N, Trevor A (1986). Interactions of the neurotoxic amine 1-methyl-4-phenyl-1,2,3,6-tetrahydropyridine with monoamine oxidases. Biochem J.

[107] Solano SM, Miller DW, Augood SJ, Young AB, Penney JB (2000). Expression of alpha-synuclein, parkin, and ubiquitin carboxy-terminal hydrolase L1 mRNA in human brain: genes associated with familial Parkinson's disease. Ann Neurol.

[108] Spillantini MG, Crowther RA, Jakes R, Hasegawa M, Goedert M (1998). Alpha-Synuclein in filamentous inclusions of Lewy bodies from Parkinson's disease and dementia with lewy bodies. Proc Natl Acad Sci U S A.

[109] Spillantini MG, Schmidt ML, Lee VM, Trojanowski JQ, Jakes R, Goedert M (1997). Alpha-synuclein in Lewy bodies. Nature.

[110] Stefanova N, Fellner L, Reindl M, Masliah E, Poewe W, Wenning GK (2011). Toll-like receptor 4 promotes α-synuclein clearance and survival of nigral dopaminergic neurons. Am J Pathol.

[111] Stefanova N, Georgievska B, Eriksson H, Poewe W, Wenning GK (2012). Myeloperoxidase inhibition ameliorates multiple system atrophy-like degeneration in a transgenic mouse model. Neurotox Res.

[112] Stefanova N, Kaufmann WA, Humpel C, Poewe W, Wenning GK (2012). Systemic proteasome inhibition triggers neurodegeneration in a transgenic mouse model expressing human α-synuclein under oligodendrocyte promoter: implications for multiple system atrophy. Acta Neuropathol.

[113] Stefanova N, Puschban Z, Fernagut PO, Brouillet E, Tison F, Reindl M (2003). Neuropathological and behavioral changes induced by various treatment paradigms with MPTP and 3-nitropropionic acid in mice: towards a model of striatonigral degeneration (multiple system atrophy). Acta Neuropathol.

[114] Stefanova N, Reindl M, Neumann M, Haass C, Poewe W, Kahle PJ, Wenning GK (2005). Oxidative stress in transgenic mice with oligodendroglial alpha-synuclein overexpression replicates the characteristic neuropathology of multiple system atrophy. Am J Pathol.

[115] Stefanova N, Reindl M, Neumann M, Kahle PJ, Poewe W, Wenning GK (2007). Microglial activation mediates neurodegeneration related to oligodendroglial alpha-synucleinopathy: implications for multiple system atrophy. Mov Disord.

[116] Su X, Maguire-Zeiss KA, Giuliano R, Prifti L, Venkatesh K, Federoff HJ (2008). Synuclein activates microglia in a model of Parkinson's disease. Neurobiol Aging.

[117] Tanji K, Odagiri S, Maruyama A, Mori F, Kakita A, Takahashi H, Wakabayashi K (2013). Alteration of autophagosomal proteins in the brain of multiple system atrophy. Neurobiol Dis.

[118] Tran HT, Chung CH, Iba M, Zhang B, Trojanowski JQ, Luk KC, Lee VM (2014). Α-synuclein immunotherapy blocks uptake and templated propagation of misfolded α-synuclein and neurodegeneration. Cell Rep.

[119] Tsuchiya K, Ozawa E, Haga C, Watabiki S, Ikeda M, Sano M (2000). Constant involvement of the Betz cells and pyramidal tract in multiple system atrophy: a clinicopathological study of seven autopsy cases. Acta Neuropathol.

[120] Ubhi K, Lee PH, Adame A, Inglis C, Mante M, Rockenstein E (2009). Mitochondrial inhibitor 3-nitroproprionic acid enhances oxidative modification of alpha-synuclein in a transgenic mouse model of multiple system atrophy. J Neurosci Res.

[121] Ubhi K, Low P, Masliah E (2011). Multiple system atrophy: a clinical and neuropathological perspective. Trends Neurosci.

[122] Ubhi K, Rockenstein E, Mante M, Inglis C, Adame A, Patrick C (2010). Neurodegeneration in a transgenic mouse model of multiple system atrophy is associated with altered expression of oligodendroglial-derived neurotrophic factors. J Neurosci.

[123] Valera E, Monzio Compagnoni G, Masliah E (2016). Review: novel treatment strategies targeting alpha-synuclein in multiple system atrophy as a model of synucleinopathy. Neuropathol Appl Neurobiol.

[124] Valera E, Spencer B, Fields JA, Trinh I, Adame A, Mante M (2017). Combination of alpha-synuclein immunotherapy with anti-inflammatory treatment in a transgenic mouse model of multiple system atrophy. Acta Neuropathol Commun.

[125] Valera E, Spencer B, Mott J, Trejo M, Adame A, Mante M (2017). MicroRNA-101 modulates autophagy and Oligodendroglial alpha-Synuclein accumulation in multiple system atrophy. Front Mol Neurosci.

[126] Vieira BDM, Radford RA, Chung RS, Guillemin GJ, Pountney DL (2015). Neuroinflammation in multiple system atrophy: response to and cause of α-Synuclein aggregation. Front Cell Neurosci.

[127] Wang W, Perovic I, Chittuluru J, Kaganovich A, Nguyen LT, Liao J (2011). A soluble α-synuclein construct forms a dynamic tetramer. Proc Natl Acad Sci U S A.

[128] Watanabe H, Saito Y, Terao S, Ando T, Kachi T, Mukai E (2002). Progression and prognosis in multiple system atrophy: an analysis of 230 Japanese patients. Brain.

[129] Wenning GK, Tison F, Ben Shlomo Y, Daniel SE, Quinn NP (1997). Multiple system atrophy: a review of 203 pathologically proven cases. Mov Disord.

[130] Wilkins A, Majed H, Layfield R, Compston A, Chandran S (2003). Oligodendrocytes promote neuronal survival and axonal length by distinct intracellular mechanisms: a novel role for oligodendrocyte-derived glial cell line-derived neurotrophic factor. J Neurosci.

[131] Woerman AL, Kazmi SA, Patel S, Aoyagi A, Oehler A, Widjaja K (2018). Familial Parkinson's point mutation abolishes multiple system atrophy prion replication. Proc Natl Acad Sci U S A.

[132] Woerman AL, Kazmi SA, Patel S, Freyman Y, Oehler A, Aoyagi A (2018). MSA prions exhibit remarkable stability and resistance to inactivation. Acta Neuropathol.

[133] Yamasaki TR, Holmes BB, Furman JL, Dhavale DD, Su BW, Song ES (2019). Parkinson's disease and multiple system atrophy have distinct α-synuclein seed characteristics. J Biol Chem.

[134] Yazawa I, Giasson BI, Sasaki R, Zhang B, Joyce S, Uryu K (2005). Mouse model of multiple system atrophy alpha-synuclein expression in oligodendrocytes causes glial and neuronal degeneration. Neuron.

[135] Zhao Q, Yang X, Tian S, An R, Zheng J, Xu Y (2016). Association of the COQ2 V393A variant with risk of multiple system atrophy in east Asians: a case-control study and meta-analysis of the literature. Neurol Sci.

